# Intraocular Pressure Changes: An Important Determinant of the Biocompatibility of Intravitreous Implants

**DOI:** 10.1371/journal.pone.0028720

**Published:** 2011-12-14

**Authors:** Ling Zou, Ashwin Nair, Hong Weng, Yi-Ting Tsai, Zhibing Hu, Liping Tang

**Affiliations:** 1 Joint Program in Bioengineering, University of Texas – Southwestern Medical Center at Dallas and The University of Texas at Arlington, Arlington, Texas, United States of America; 2 Departments of Physics and Materials Science and Engineering, University of North Texas, Denton, Texas, United States of America; Instituto de Engenharia Biomédica, University of Porto, Portugal

## Abstract

**Background:**

In recent years, research efforts exploring the possibility of using biomaterial nanoparticles for intravitreous drug delivery has increased significantly. However, little is known about the effect of material properties on intravitreous tissue responses.

**Principal Findings:**

To find the answer, nanoparticles made of hyaluronic acid (HA), poly (l-lactic acid) (PLLA), polystyrene (PS), and Poly N-isopropyl acrylamide (PNIPAM) were tested using intravitreous rabbit implantation model. Shortly after implantation, we found that most of the implants accumulated in the trabecular meshwork area followed by clearance from the vitreous. Interestingly, substantial reduction of intraocular pressure (IOP) was observed in eyes implanted with particles made of PS, PNIPAM and PLLA, but not HA nanoparticles and buffered salt solution control. On the other hand, based on histology, we found that the particle implantation had no influence on cornea, iris and even retina. Surprisingly, substantial CD11b+ inflammatory cells were found to accumulate in the trabecular meshwork area in some animals. In addition, there was a good relationship between recruited CD11b+ cells and IOP reduction.

**Conclusions:**

Overall, the results reveal the potential influence of nanoparticle material properties on IOP reduction and inflammatory responses in trabecular meshwork. Such interactions may be critical for the development of future ocular nanodevices with improved safety and perhaps efficacy.

## Introduction

Posterior ocular diseases, including glaucoma, macular degeneration, uveal melanoma and retinoblastoma are often hard to be treated due to ocular tissue barriers [1]–[3]. While topical administration is effective in the treatment of anterior chamber diseases, it is ineffective in the treatment of diseases afflicting the posterior segments of the eye [2]. Major problems include washing away of the drug by tears and the inefficient diffusion of drug from the corneal side to the posterior [4], [5]. Systemic injection does deliver drugs to the posterior of the eye but is also associated with non-specific accumulation of drug in other organs. In addition the blood retinal barrier also hinders the diffusion of drug into the posterior chamber [2]. In light of this information, intraocular drug injections have gained in importance. However, although they achieve therapeutic drug levels, they are associated with high vitreal clearance which necessitates multiple injections. This in turn leads to complications of endophthalmitis and retinal detachment [2], [3], [6]. There is a need for the development of alternative treatments for posterior ocular diseases.

Many therapeutic strategies have been developed in recent years. One such method is the use of biomaterial drug delivery devices either in the form of implants or as micro or nanoparticles [2], [7], [8]. Despite of their ability to release therapeutic agents for a prolonged period of time, ocular rod implants have been found to be responsible for causing retinal detachment and endophthalmitis [6]. With the expansion of nanotechnology in medicine, a wide variety of nanoparticle drug releasing devices have been fabricated and tested for their ability to treat a wide range of diseases [1], [9]–[12]. Many studies have been done to explore the possibility of using polymeric micro and nanoparticles for anterior and posterior chamber drug delivery [1], [9]–[15]. Although microparticles have better drug loading capacity than nanoparticles, the latter is recognized as favorable drug carrier due to its low risk on hampering normal vision [16], [17]. Although different types of nanoparticles have been investigated for their ability to target different cells, tissues and to cure different ocular diseases [9]–[12], [14], [15], [18]–[22]. very limited studies have been done to systematically evaluate the effect of material physical and chemical properties on their ocular tissue and cell compatibility.

It is well established that the physical and chemical properties of materials affect their cell and tissue compatibility [23]–[27]. We thus assumed that nanoparticles made of different materials are likely to cause different extents of acute tissue responses in the eye. To test this hypothesis, nanoparticles made of different materials were included in this study. Specifically, nanoparticles were made out of degradable polymers like poly (l-lactic acid) (PLLA), hydrogels like poly N-isopropyl acrylamide (PNIPAM), non-degradable materials like polystyrene (PS), and biological materials like hyaluronic acid (HA). The ocular compatibility of these nanoparticles was evaluated using rabbit intravitreous implantation model. After implantation for different periods of time, we measured the changes in intraocular pressure (IOP). At the end of the studies, animals were sacrificed and ocular tissues were histologically evaluated. The effect of material properties on the ocular tissue responses was then determined to show that it can play a key role in determining the fate of nanoparticles in the eye.

## Methods

### Ethics Statement

The animal use protocols (A06-028, A09-028) were reviewed and approved by the Institutional Animal Care and Use Committee of the University of Texas at Arlington.

### Materials

N-Isopropylacrylamide was purchased from Polysciences. Sodium acrylate (NaAc), N,N′-Methylene-bis-acrylamide (BIS) and potassium persulfate (KPS) were purchased from Bio-Rad (Hercules, CA, USA). Poly (L-lactic acid) (PLLA, MW 1.37×10^6^ kDa) was purchased from Birmingham Polymers (Birmingham, AL, USA). N-(3 Dimethylaminopropyl)-N′-ethylcarbodiimide hydrochloride (EDAC), Fluorescein isothiocyanate (FITC), Polystyrene and hyaluronic acid were purchased from Sigma, St. Louis, USA. Balanced Salt Solution (BSS) was obtained from Alcon Inc (Fort Worth, TX, USA).

### Nanoparticle synthesis

Both poly N-isopropylacrylamide (PNIPAM) and PS nanoparticles were synthesized based on established techniques [28], [29], [30]–[32]. To study the distribution of the intravitreous injected nanoparticles, some PNIPAM nanoparticles were conjugated with FITC using carbodiimide chemistry [33], [34]. HA nanoparticles were synthesized as described earlier [35]. Briefly, acetone was added in a weight ratio of 100∶80 to a 0.5 wt% HA solution and the HA/water/acetone mixture was stirred for 2 hours. EDAC was added to the mixture in a weight ratio of 0.05∶100 to form a crosslinked mixture. This mixture was then stirred at 20 to 22°C for approximately 24 hours after which acetone in a weight ratio of approximately 60∶100 was added to form the final mixture that was stirred for 20 hours and dialyzed against distilled water to form HA nanoparticles.

### Animal implantation

Dutch rabbits (4–5 lb) were purchased from Myrtle's Rabbitry Inc (Thompsons Station, TN). All experimental procedures were approved by the Institutional Animal Care and Use Committee (IACUC) at the University of Texas at Arlington and carried out under veterinary supervision. Prior to the procedures, animals were sedated by subcutaneous injection of a 1∶5 mixture of 100 mg/ml xylazine (Rompun; Miles Laboratories, Shawnee Mission, KS) and 100 mg/ml Ketamine HCl (Ketaset; Bristol laboratories. Syracuse, NY). One drop of topical anesthetic Proparacaine HCl (0.5%; Alcon Laboratories, Fort Worth, TX) was administered to each eye before injection. One hundred µl (20 g/l) of each type of particle was injected in to intravitreal space of the right eye via 30 gauge needle on a 1 ml tuberculin syringe. The intravitreal space of the left eye was injected with 100 µl of balanced salt solution (BSS) and served as control. The points where the intravitreal injections were made were approximately 2–3 mm from the corneal limbus as suggested in early publications [36], [37]. All injections were performed by the same researcher to avoid individual operational variations.

### Intraocular pressure measurement

Intraocular pressure (IOP) has often been used as an indicator for various ocular diseases [38], [39]. The IOP was measured using a calibrated pneumatonometer (Model 30 Classic; Mentor Co., Norwell, MA, U.S.A.) one day before and daily for 3 days after nanoparticle injection as described earlier [40]. The results of the IOP reading were taken in the confidence interval greater than or equal to 95%. Measurements were taken at the same hour in order to avoid circadian changes. The changes in IOP were calculated by subtracting the IOPs at the end of 3 days from that before particle injection.

### Ocular imaging and histological Analyses

After intravitreous implantation of nanoparticles for different periods of time (2 hrs, 4 hrs and 1 day), rabbits were euthanized and eyes were recovered and frozen sectioned. To image intravitreous distribution of nanoparticles, ocular tissue sections from animals implanted with PNIPAM nanoparticles were scanned using a Genepix Microarray analyzer (Molecular Devices, Sunnyvale, CA). The fluorescence intensities in different areas of the ocular tissues were analyzed and then compared. Furthermore, some tissue sections were H&E stained and the extent of implant-associated ocular tissue responses was quantified by measuring the thickness of various ocular tissues like cornea, iris and retina using a Leica microscope (Leica Microsystems, Wetzlar, GmbH) equipped with a Nikon E500 Camera (8.4 V, 0.9 A, Nikon Corp., Japan). To assess the extent of acute inflammatory responses, some tissue sections underwent immunohistological staining for CD11b+ inflammatory cells in which images were captured with a CCD camera (Retiga EXi, Qimaging, Surrey BC, Canada) and cell densities were analyzed using NIH ImageJ program as described in our previous publications [41], [42].

### Statistical Analyses

All results were expressed as mean ± SD. All statistical comparisons were made with BSS controls using t-tests. Differences were considered statistically significant at p<0.05 Linear regression analyses was conducted with the Intraocular pressure change represented by mm of Hg was used as the dependent variable, while the CD11b+ cell numbers were the explanatory variables.

## Results

### Changes in intraocular pressure after intravitreous particle injection

**Figure 1:**
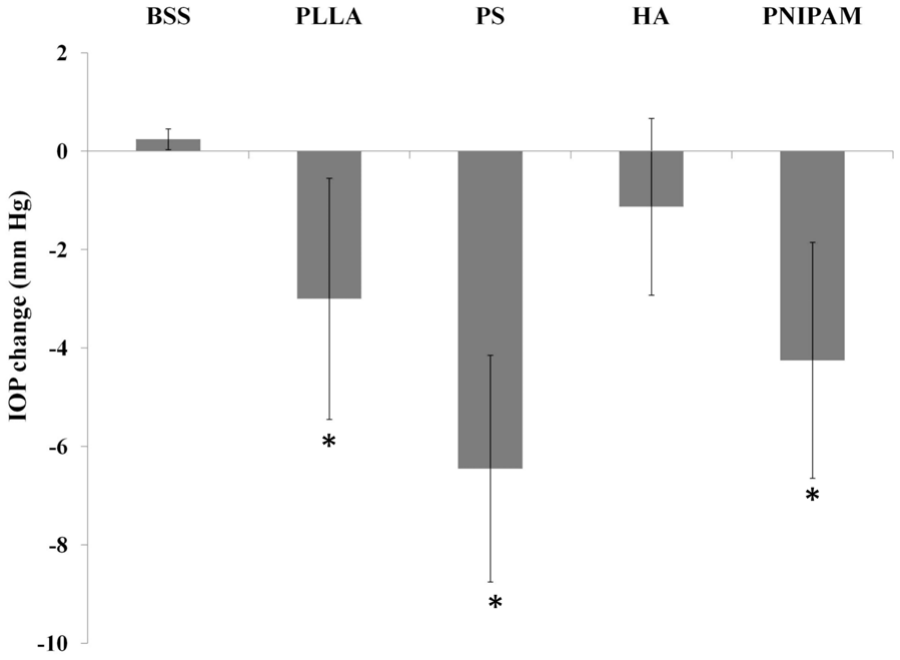
Assessment of mean intraocular pressure variations after administration of particles made of poly-L lactic acid (PLLA), polystyrene (PS), hyaluronic acid (HA), and poly N-isopropyl acrylamide (PNIPAM) and Balanced Salt Solution (BSS) in the vitreous. Data are mean ± standard deviation. Significance of PLLA, PS, HA, PNIPAM vs. BSS control; * p<0.05.

Particles made of different materials were used in this investigation (Table 1). These particles had diameter between 100–200 nm. Both PLLA and PS particles are hydrophobic while HA and PNIPAM particles were hydrophilic. It should be noted that PLLA and HA particles are biodegradable whereas PS and PNIPAM particles are non-degradable. Following intravitreous implantation, nanoparticle implants did not trigger apparent adverse response or abnormality. Rather surprisingly, we found that the intravitreous implantation of particles affected IOP significantly. Specifically, the injection of PLLA, PS and PNIPAM particles caused substantial reduction of IOP (Figure 1). On the other hand, the injection of HA nanoparticles and BSS (as control) had no effect on IOP. Such pressure changes lasted for less than 3 days. Although the cause of such particle implant-mediated IOP reduction was not known, it is likely that the material property of particle implants affects their acute ocular compatibility.

**Table 1:** Physical properties of nanoparticles used for intravitreous implantation.

Material	Type	Wettability	Degradable	Size nm
Buffered Salt Solution	*Solution*	Aqueous solution	NA	NA
Poly (l-lactic) Acid	*Polymeric*	Hydrophobic	Yes	143
Polystyrene	*Polymeric*	Hydrophobic	No	100
Hyaluronic Acid	*Polymeric*	Hydrophilic	Yes	200
Poly N-isopropyl acrylamide	*Polymeric Hydrogel*	Hydrophilic	No	100

### Distribution of intra-vitreous injected nanoparticles

**Figure 2:**
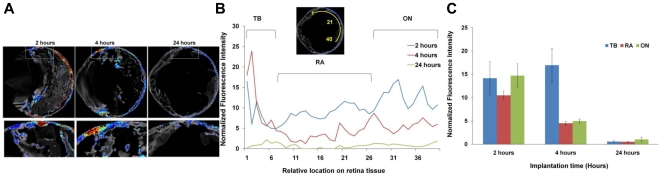
Distribution of FITC-labeled PNIPAM nanoparticles after intravitreous administration over a day. Localization of fluorescence in the posterior segments of the eye at various time points (2, 4 and 24 hours) was observed (A) and quantified (B). Quantification of the normalized fluorescence intensity at various locations (TB: Trabecular Meshwork; RA: Retina; ON: Optic Nerve) at various time points (C).

To determine the potential interaction between injected particles with ocular tissues, we first monitored the particle distribution following intravitreous implantation using FITC-labeled PNIPAM particles. As shown in the whole ocular section images, we found that, at 2 hours, implanted particles were only found in the posterior, but not anterior segment of eye. We also found uniform fluorescence along the wall of the posterior segments indicating the even spread of particles over the retinal tissue (Figure 2A). Interestingly, we found that substantially more particles accumulated in the trabecular meshwork area even at an early time point – 2 hours. With increasing amount of time (4 and 24 hours) following implantation, we found that the particle-associated fluorescence intensities reduced substantially in the posterior chamber (Figure 2A and B). Interestingly, by 4 hours the fluorescence intensity at the trabecular meshwork was significantly higher than the rest of the eye and substantial fluorescent signals were also found outside the ocular tissue nearby the trabecular outflow region. Based on the fluorescent intensity measurements and distribution, noticeably, most of the particles cleared from the central portion of posterior chambers while majority of the residual fluorescence intensity was seen in the area of trabecular meshwork prior to clearance from the posterior cavities shortly after 24 hours. Quantification of the distribution of fluorescent particles throughout the ocular tissues further confirmed the presence of particles in the trabecular meshwork area (Figure 2C). These results suggest that the particle implants have little or no contact with corneal and iris tissues. On the other hand, based on the fluorescence distribution, it is likely that many posterior ocular tissues, including retina and trabecular meshwork, were exposed to particle implants.

### Effect of material properties on ocular tissue responses

**Figure 3:**
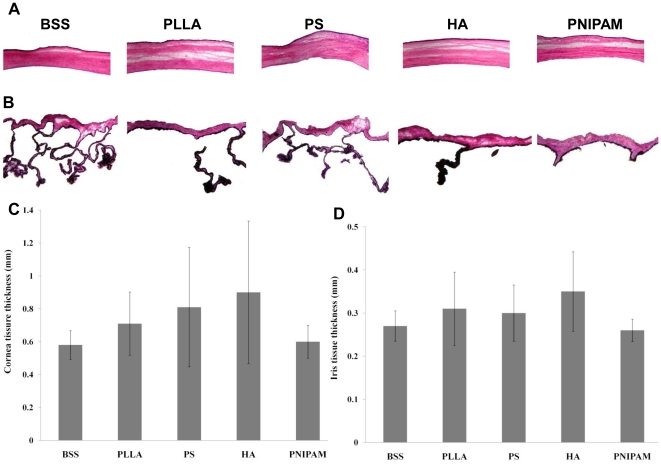
Histological assessment of corneal and iris tissue after intravitreous implantation. The representative H&E images of the cornea (A) and iris (B) tissue were shown here. Based on H&E staining images, the influence of particle property on the thickness of the corneal (C) and iris (D) thickness were quantified. Data are mean ± standard deviation. Significance of PLLA, PS, HA, PNIPAM vs. BSS control; * p<0.05.

**Figure 4:**
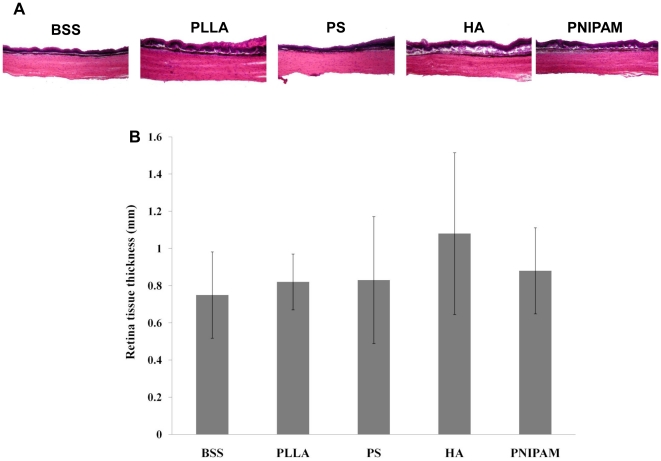
Evaluation of retinal tissue morphology following intravitreal injection. Representative H&E stained retinal tissues were shown here (A). The thickness of the retinal tissue was also calculated and then compared (B). Data are mean ± standard deviation. Significance of PLLA, PS, HA, PNIPAM vs. BSS control; * p<0.05.

To assess the potential ocular compatibility of particle implants, various ocular tissues in both anterior and posterior segments were histologically analyzed. As expected, we found that the intravitreous injection of particles have no apparent influence on the anatomical structure of cornea (Figure 3A) and iris (Figure 3B) tissue based on morphological assessment of tissue thickness (Figures 3C & D). Although PNIPAM particle-implanted animals showed the lowest corneal and iris tissue thickness, the differences were not statistically significant as compared with those in animals injected with BSS control. Rather surprisingly, despite of the apparent interaction between particles and retinal tissue, we found that particle implants have no influence on the anatomical structure and thickness of retinal tissues (Figure 4A & B). How the injection of particle implants reduced IOP, was yet to be answered.

### CD11b+ Inflammatory cell accumulation in trabecular meshwork tissue

**Figure 5:**
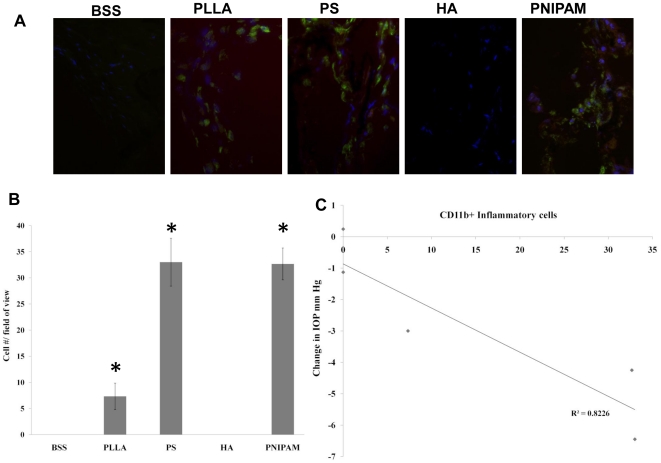
The accumulation of CD11b+ inflammatory cells in the trabecular meshwork. The representative immunohistochemically stained images showed the accumulation of CD11b+ inflammatory cells (labeled green; DAPI staining to locate cell nucleus) in the trabecular meshwork following particle injection (A). The extent of CD11b+ cell accumulation in the trabecular meshwork was quantified (B). Data are mean ± standard deviation. Significance of PLLA, PS, HA, PNIPAM vs. BSS control; * p<0.05 Correlation between CD11b+ inflammatory cells and average IOP changes in different test groups was also determined (C).

It is well established that trabecular meshwork is responsible for controlling IOP [43], and increased inflammatory responses in trabecular meshwork have been shown to cause IOP reduction in human patients [44]. Since our results have shown that substantial portion of the injected particles accumulated in trabecular meshwork prior to clearance from the posterior chamber, we assumed that some particle implants may trigger such inflammatory responses in trabecular meshwork tissue and then lead to the reduction of IOP. To test this hypothesis, immunohistological analysis of ocular tissues was performed by examining the presence or absence of CD11b+ inflammatory cells in the trabecular meshwork area around the ciliary body. Indeed, we found that both PS and PNIPAM particle groups were associated with very high CD11b+ cell accumulation (labeled green) (Figure 5A) while PLLA particles prompt less (∼20%) CD11b+ cell accumulation as compared with PS and PNIPAM particle groups. It should be noted that there were almost no CD11b+ cells in the HA nanoparticle group as well as BSS control. The average CD11b+ cell numbers from numerous sections were quantified to substantiate the visual observations (Figure 5B). To investigate the relationship between inflammatory cell accumulation and IOP reduction, then numbers of CD11b+ cells from different sample groups were then correlated with averages of IOP from respective sample groups. As expected, there was a very good relationship between CD11b+ cell numbers found in the trabecular meshwork tissues and overall IOP reduction (Figure 5C).

## Discussion

Drug delivery to the back of the eye, especially the posterior segments, is a key research area and important considering numerous ocular diseases that afflict that region. However, most drug administration techniques like topical administration or even implantable rods have their own drawbacks as has been reviewed in our earlier publication [3]. Administration of drug directly into the vitreous is also associated with a lot of problems; clearance of the drug being one of the main drawbacks [2], [45]. In light of this fact, nanotechnology has come to the forefront of ocular drug delivery and as such, understanding ocular tissue response to implanted nano-biomaterials is of paramount significance [2], [3], [8], [46]. Our selection of materials to synthesize various nanoparticles was based on the following facts. First, a vast majority of drug releasing nanodevices are made out of FDA approved polymers like PLLA and PLGA and are usually in the range of 10 nm to 1000 nm [2], [8], [47]. In fact, previous studies have shown that particles in the range of 20 nm to 200 nm have the highest affinity to tissue [13], [48]. Second, hydrogels like PNIPAM have been extensively researched for drug delivery applications [14], [49]–[51]. Thirdly, HA is a major component of the vitreous and as such, their presence in the eye should be tolerable. HA makes up a sizeable proportion in the retinal pigment epithelium and interphotoreceptor matrix [52]. Considering this information, we selected nanoparticles made out of PLLA, PS, HA and PNIPAM in the size range of 100 to 200 nm.

It is well established that material properties trigger different extent of soft tissue responses [25], [53]. We thus assumed that material properties would exert some influence on ocular tissue reactions. Very few studies have been done to assess the effect of material properties on ocular compatibility of particles. Nevertheless, studies have found that PLLA and PLGA nanoparticles can be used for delivery of high molecular weight drugs to the retina [54], and poly (ε-caprolactone) is well tolerated by retinal tissue for at least 4 weeks [55]. Non-toxic chitosan and hyaluronic acid have been found to be good drug carriers [56], [57], and carbodiimide crosslinked hyaluronic acid has been shown to have good ocular compatibility in the anterior chamber [58]. PNIPAM hydrogel grafted with chitosan has also been applied as a thermally responsive ophthalmic drug delivery device [51]. Also most of the studies until now have mainly focused on visual signs of inflammation to suggest lack of biocompatibility.

To determine the ocular tissue responses to particle implants, we first measured the IOP changes following intravitreous implantation of particle. The fluctuation of IOP indicates the balance between production and drainage of aqueous humor and hence it was measured to determine the impact of various nanoparticle injections on aqueous humor drainage [43], [59]. In addition, it has been documented that ocular inflammation strongly influences the IOP [60]–[63]. Diseases like glaucoma have been shown to increase IOP while inflammatory conditions produced by anterior uveitis and iritis were found to reduce IOP [44], [64]–[67]. Substantial studies in glaucoma research have focused on using various pharmacological approaches to reduce IOP for prolonged period of time [64], [68]. Interestingly, our studies have found that the intravitreous implantation of particles prompted different extent of IOP reduction. Specifically, we found that PNIPAM and PS particles triggered the maximum reduction in IOP, while PLLA particles caused a rather mild reduction in IOP. Most interestingly, our results show that the implantation of HA particles trigger minimal or no IOP reduction. In fact, many studies have shown that HA particles have superb tissue compatibility in other body parts [56], [58], [69], [70], in different animal models [70], [71]. In fact a study evaluating the biocompatibility of intravitreal hyaluronic acid implants found that there were no evident signs of inflammation following implantation [71]. These results suggest that HA particles are good nanocarriers for posterior drug delivery.

To find the cause for particle implant-mediated IOP reduction, we first observed the distribution of the implants following intravitreous implantation. Although extensive research efforts have been placed on the development of nanocarriers for anterior and posterior ocular drug delivery, little has been done to study the fate of particle implants following injection. Briefly, these studies have revealed that nano and microparticles can reach the intraocular tissues when administered systemically or through periocular administration routes [48], [72]. However, it has also been reported that systemic administration of drug requires high doses to offset loss due to non-specific targeting and systemic side effects [73]. Our studies revealed that, rather surprisingly, majority of the particle implants injected intravitreally, only stay in the posterior segments for a very short period of time (∼24 hours). These results support that that, differing from common assumption that intravitreous administration will lead to better distribution, humor flow actively pushed particle implants out of the posterior. In addition, particle implants may reach retinal tissues shortly after intravitreous injection. Although the fate of the particles is yet to be determined, it is plausible that most of the particle implants exit the posterior chamber via the trabecular meshwork based on the fluorescent intensity distribution. This observation is supported by several earlier observations that particles and drugs may leave vitreous compartment via the trabecular meshwork [43], [74].

Particle implants have been shown to trigger immune reactions in the surrounding tissues [23], [25], and it is likely that similar particle-mediated tissue responses are also found in ocular tissues. To the best of our knowledge, very few studies have been done in this regard. A few studies have tested poly(ortho esters) following intravitreal injection and found no evident signs of inflammatory reaction up to 3 months [75]. Studies involving PLGA microspheres [76], and porous silicon microparticles [77], found that they were well tolerated with no clinical signs of inflammation based on visual examination even four days to four months after implantation. A recent study has also evaluated the ocular compatibility of gluteraldehyde crosslinked and EDAC crosslinked hyaluronic acid implants in the anterior chamber and found that EDAC crosslinked implants were more compatible [58]. However, it is mostly unclear whether the intravitreous implantation of particles would trigger immune reactions in different ocular tissues, including cornea, iris, retina and trabecular meshwork. To our surprise, we found that all the tested particle implants had no significant influence on the morphology and anatomical structure of cornea, iris, retina tissue despite of apparent short term accumulation of particle implants in nearby retinal tissue.

It is well established that trabecular meshwork and surrounding ciliary body is responsible for maintaining the IOP [78]–[81]. To search for the cause of IOP changes following particle implantation, we examined the tissue responses in the trabecular meshwork. It should be noted that the potential influence of intravitreous particle implants on trabecular meshwork function have not been evaluated prior to this work. The trabecular meshwork, upon examination showed signs of apparent acute inflammatory responses with accumulation of CD11b+ cells in some groups of animals, especially animals with PS particle or PNIPAM implants. Mild accumulation of CD11b+ cells in trabecular meshwork was also found in animals implanted with PLLA particles. Interestingly, no CD11b+ cells were found in the tissues isolated from animals implanted with either HA particles or BSS controls. Equally importantly, we found a good relationship between the numbers of CD11b+ cells in trabecular meshwork and the average IOP reduction in different groups of implants. These results suggest that particle implant-associated inflammatory responses in trabecular meshwork are responsible for IOP reduction and are supported by many earlier works in which inflammatory responses inside trabecular meshwork have been linked to the reduction of IOP [44], [65], [82].

The results from this study have emphasized the fact that IOP should be measured as part of the evaluation of tissue compatibility of ocular implants, specifically in the case of nanoparticle and microparticle implants. Furthermore, the “normal” anatomical structure of retinal, corneal and iris tissue does not guarantee the safety of ocular particle implants. Rather, the histological evaluation of inflammatory responses in trabecular meshwork should be done as an indicator of ocular compatibility of intravitreous implants. Finally, further studies are needed to investigate the influence of material physical and chemical properties on IOP changes and on trabecular tissue responses.
